# Nitrogen enrichment enhances the negative top–down effect on plant functional traits

**DOI:** 10.3389/fpls.2024.1418724

**Published:** 2024-08-30

**Authors:** Dongmei Zhang, Liwen Zhang, Siqun Lan, Lianjun Zhao, Guangxuan Han, Lin Chen

**Affiliations:** ^1^ Tianjin Key Laboratory of Animal and Plant Resistance, College of Life Sciences, Tianjin Normal University, Tianjin, China; ^2^ CAS Key Laboratory of Coastal Environmental Processes and Ecological Remediation, Yantai Institute of Coastal Zone Research (YIC), Chinese Academy of Sciences (CAS), Shandong Key Laboratory of Coastal Environmental Processes, YICCAS, Yellow River Delta Field Observation and Research Station of Coastal Wetland Ecosystem, YICCAS, Yantai, China; ^3^ School of Resources and Environmental Engineering, Ludong University, Yantai, China; ^4^ College of Environment and Planning, Liaocheng University, Liaocheng, China

**Keywords:** nitrogen addition, top-down effect, functional traits, Yellow River delta, common reed

## Abstract

Eutrophication resulting from anthropogenic activities has been recognized as a significant driver of changes in ecosystem functioning. Furthermore, it may exacerbate the top–down effect and thus exert an important impact on plant growth. To test this hypothesis, we conducted a 3-year manipulative field experiment to investigate the impacts of nitrogen addition and crab herbivory on the growth of *Phragmites australis* in the salt marsh of the Yellow River Delta. The results demonstrated that a 3-year nitrogen addition can significantly increase the total nitrogen and carbon content of *P. australis* leaves, thereby enhancing their nutritional value and palatability, as well as increasing the proportion of leaves consumed by crabs. Therefore, nitrogen addition together with crab herbivory had a significant negative effect on *P. australis* height, leaf length, and leaf breadth in the ambient crab and procedural crab cage treatment compared to the crab exclusion treatment. The structural equation modeling further substantiated these findings. The model revealed a direct and positive correlation between nitrogen addition and leaf nutrient content (path coefficient = 0.34). Additionally, it demonstrated a direct and positive relationship between leaf nutrient content and the proportion of leaves consumed by crabs (path coefficient = 0.22). Simultaneously, there was an observed negative correlation (path coefficient = − 0.37) between the proportion of leaves consumed by crabs and plant functional traits, represented by leaf length in the model, during 2018. Moreover, the crab exclusion treatment significantly reduced the proportion of leaves consumed by crabs and thus enhanced the *P. australis* individuals, leaf number, and biomass. Overall, crab herbivory had a significant detrimental top–down effect on the growth of *P. australis*, and nitrogen enrichment may exacerbate this top–down effect. The findings of our study highlight the combined adverse effects of nutrient enrichment and top–down on plant functional traits and plant growth. The findings of this study will contribute to a comprehensive understanding of the underlying factors influencing vegetation degradation in coastal wetland, thereby establishing a solid theoretical framework for the conservation and management of wetland ecosystems within the context of global environmental change.

## Introduction

Eutrophication, as one of the most significant drivers of global change, has at least doubled the supply of nutrients to the biosphere (He and Silliman, 2015). Numerous studies have shown that increases in nutrient content can have far-reaching effects on both producers and consumers. Tidal wetlands as important regulators of nearshore water quality are also susceptible to eutrophication, and nutrient overenrichment can affect a range of functional and structural characteristics of tidal wetlands and their species, including primary production, consumer activities, carbon sequestration, competitive hierarchy, and species composition (Bertness et al., 2008, Bertness et al., 2009; Valdez et al., 2023).

The regulation of ecological processes is also significantly influenced by top–down effects, wherein herbivory is a mechanism for energy transfer from primary producers to higher trophic levels and a key mechanism for regulating plant survival, growth, reproduction, population dynamics, and community composition (Poore et al., 2012; Jia et al., 2018; Barnes et al., 2020). For terrestrial ecosystems, the exclusion of invertebrate herbivores was found to result in a decrease in plant species richness and an increase in biomass, indicating that herbivores can affect plant diversity by altering competitive interactions between species (Allan and Crawley, 2011; Filazzola et al., 2020; Wang et al., 2023). In the investigation of marine ecosystems, top–down effects were found to be predominant in coral reef systems, while in wetland ecosystems, consumers were found to exert a significant inhibitory effect on the survival, growth, and reproduction of salt marsh plants (Alberti et al., 2011; He and Silliman, 2016). Both large herbivores (cattle and sheep) and small herbivores (insects) play an important role in plant population dynamics and community composition (La Pierre et al., 2014; Borgstrom et al., 2017).

After extensive deliberation, it is now widely acknowledged that both bottom–up effects (e.g., nutritional and physical factors) and top–down effects (e.g., herbivory) are important controls on plant productivity in numerous ecosystems, with the possibility of these two factors operating synergistically (Alberti et al., 2011; Jia et al., 2018). The addition of nutrients typically promotes plant growth and increases plant biomass, whereas the presence of herbivores reduces plant biomass (Gruner et al., 2008; Peltzer et al., 2010). The findings of numerous studies have demonstrated that the addition of nutrients, such as nitrogen, results in an increase in plant palatability, consequently leading to a subsequent increase in herbivory on the plant (Cebrian et al., 2009). However, it is important to note that this nutrient-induced promotion of plant growth may be counterbalanced by negative impacts, ultimately resulting in lower biomass under fertilized conditions compared to unfertilized conditions (Bertness et al., 2008). The coastal wetlands play a crucial role in providing various ecosystem services to human societies, including storm and hurricane protection, pollution mitigation, carbon storage, and economic benefits (Liu and Ma, 2024). However, human activities are altering shoreline evolution and wetland culture and causing detrimental impacts on ecosystem functions and services (Cui et al., 2016). Salt marshes are spatially and temporally variable transition zones between freshwater and marine environments (Rogers et al., 2024). As the more studied shoreline community, it has high productivity but has strong plant growth limiting-factors such as nitrogen effectiveness and herbivory. Therefore, investigating the interplay between bottom–up effects and top–down effects on coastal wetland vegetation holds crucial implications for the preservation of wetland ecosystems (Alberti et al., 2010; Schultz et al., 2016).

Located at the confluence of the Bohai Sea and Laizhou Bay, the Yellow River Delta (YRD) harbors extensive intertidal wetlands in the warm-temperate zone of eastern China. It serves as a crucial wintering habitat for birds, akin to numerous other coastal wetlands worldwide. The ecological functions of YRD encompass biodiversity conservation, flood and drought mitigation, climate regulation, pollution reduction, and natural disaster resilience (Zhang et al., 2021). The plant species composition is relatively simple, characterized by the dominance of mono-dominant plants and well-defined zonation. *Phragmites australis* is one of the dominant plants in YRD, accounting for about 5.39% of the YRD area (317 km^2^) (Fan et al., 2020). As the dominant vegetation type, the *P. australis* rhizome system facilitates the accumulation of pollutants and plays an important role in purifying the water quality of the wetland and maintaining the stability of the wetland ecosystem; therefore, the health of *P. australis* vegetation is the key to the stability of the wetland system (Liu et al., 2012; Rezania et al., 2019; Razieh et al., 2022; Zou et al., 2023). Previous research has primarily focused on the response of *P. australis* to salinity and water table gradients, as well as factors influencing its morphological and ecophysiological traits, population dynamics, and spatial distribution (Guan et al., 2016). However, the number of studies on the consumer impacts of *P. australis* is limited. Herbivorous crabs, as one of the dominant herbivores in the salt marshes of YRD, exert significant influence on *P. australis* growth through their consumption of *P. australis* (Zhang et al., 2021). Therefore, *P. australis* zonation in YRD is an ideal study site for investigating the interplay between bottom–up and top–down effects.

We conducted a 3-year field experiment in the salt marshes of YRD starting in May 2018. Through nitrogen addition and manipulation of herbivorous crabs (including the ambient crab, the procedural crab cage, and crab exclusion cage), the procedural crab cage was set up to provide a control against the crab exclusion treatment, ensuring that *P. australis* growth under the similar conditions remained consistent in both the presence and absence of crab herbivory. We aimed to address the following scientific questions: (1) Does crab herbivory inhibit *P. australis* growth in tidal wetlands? (2) Does nitrogen addition promote crab herbivory? (3) How does the interaction of nitrogen addition and crab herbivory affect *P. australis* growth over increasing years?

## Materials and methods

### Study site

**Figure 1:**
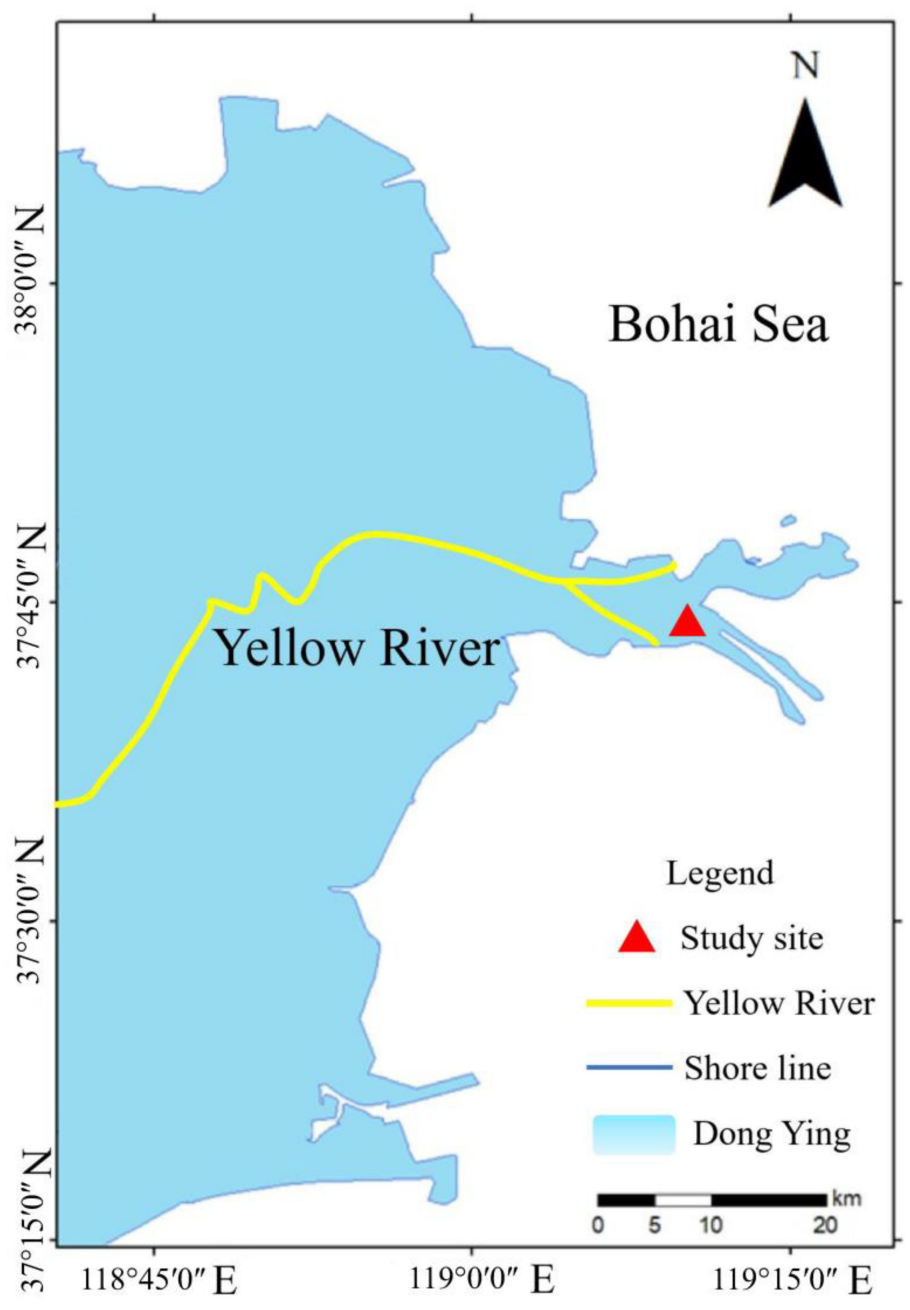
The location of the study site.

The study site located in the salt marsh of YRD in Shandong Province (37°44′5″ N, 119°12′56″ E) (**Figure 1**). The climate of this region is warm temperate, with an annual average temperature of 11.7°C–12.6°C; annual average rainfall of 530–630 mm, with much of the rainfall occurring in July and August; and annual average evaporation of 1,750–2,430 mm (Zhang et al., 2021). The dominant plants within the salt marshes of YRD show a regular zonal distribution, with species such as *Spartina alterniflora* (invasive species), *Suaeda salsa*, and *P. australis* (Zou et al., 2023). Furthermore, the dominant crab species in the studied salt marsh is *Helice tientsinensis*. This crab is an omnivorous species, and our previous study utilizing stable isotope analysis to determine food sources revealed that *P. australis* leaves were one of the important dietary components for *H. tientsinensis* in this salt marsh of YRD (Lan et al., 2020).

### Field experiment

We conducted a 3-year experiment (from May 2018 to September 2020) in the *P. australis* zone of the intertidal marsh YRD. The experiment was a completely random design with two treatments: nitrogen addition and crab herbivory, and the nitrogen addition levels were 0 (N0) and 20 g/m/year (N1). We opted for a high nitrogen addition rate due to the potential loss of nitrogen fertilizer caused by tidal action in the salt marsh. We evenly applied the nitrogen fertilizer (urea) to the nitrogen addition plots in May and July of each year.

**Figure 2:**
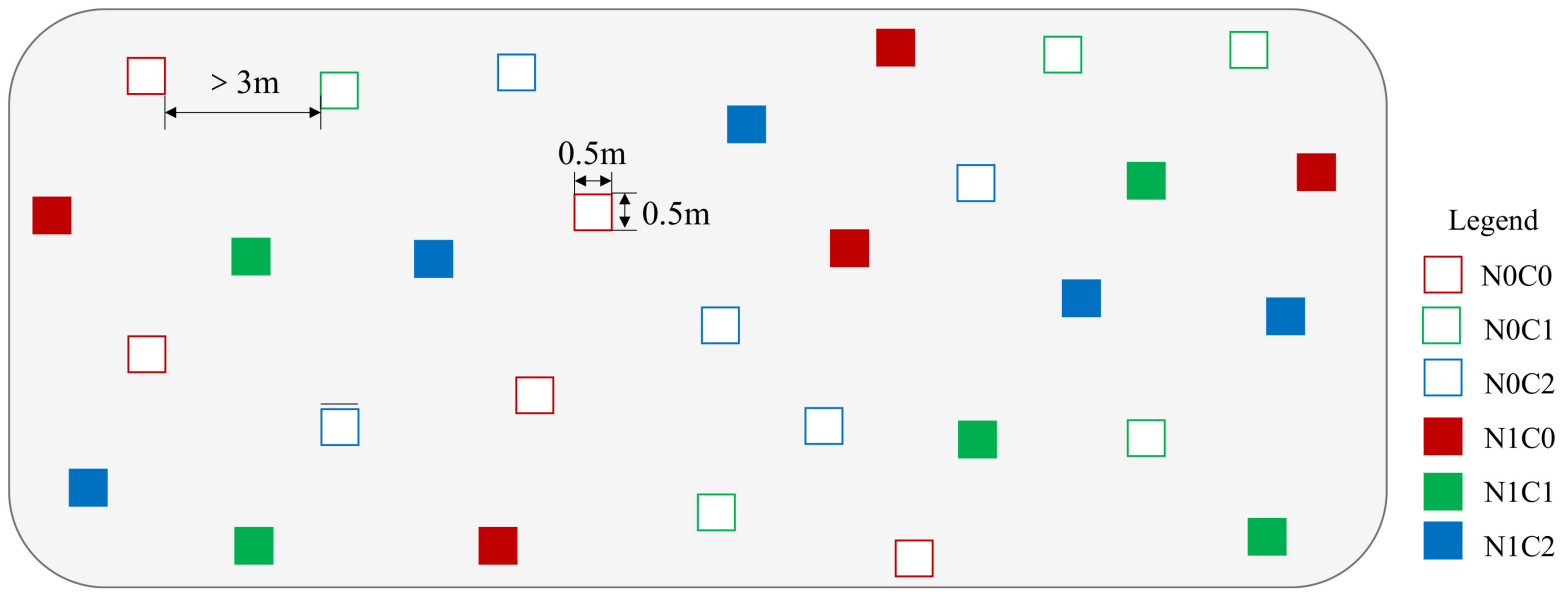
Schematic diagram of the experimental plots. N0C0, without nitrogen addition and ambient crab treatment; N0C1, without nitrogen addition and procedural crab cage treatment; N0C2, without nitrogen addition and crab exclusion treatment; N1C0, nitrogen addition and ambient crab treatment; N1C1, nitrogen addition and procedural crab cage treatment; N1C2, nitrogen addition and crab exclusion treatment.

The crab herbivory included three treatments: the ambient crab treatment (C0), the procedural crab cage treatment (C1), and the crab exclusion cage treatment (C2). Each treatment was replicated five times, resulting in a total of 30 plots (**Figure 2**). The experimental setup consisted of a rectangular frame made of PVC pipes, measuring 50 cm in length, 50 cm in width, and 150 cm in height. The crab exclusion cage was constructed with a 1-cm nylon fishing net buried 40 cm underground to prevent the crabs from entering through holes. One side of the cage served as a movable opening for conducting investigations. The procedural crab cage was similar to the crab exclusion cage but had a passageway located at the lower part that opened 10 cm from the ground, allowing crabs to pass through. In the ambient crab treatment, only PVC pipe racks were placed without attached nylon nets (**Supplementary Figure S1**). All crabs in the group were excluded by removing the crab cages after the experimental setup was completed (Zhang et al., 2021).

### Soil physical and chemical properties

During 2018–2020, at the end of the growing season (September), the soil samples were collected from the sample plots using a soil auger measuring 2 cm in diameter. Subsequently, they were air-dried under shade, followed by passing through a 100-mesh sieve. The soil pH was determined using the QT-PH220S pH meter (Beijing Channel Scientific Instrument Co. Ltd., China) with a soil-to-water ratio of 1:5. The electronic conductivity of the soil was measured by a DDBJ-350 electronic conductivity meter (Inesa Scientific Instrument Co. Ltd.; soil/water = 1:5). The Vario Micro Cube Elemental Analyzer (Elementar Analysensysteme GmbH, Hanau, Germany) was utilized for the determination of total nitrogen (TN) content in soil.

### The plant’s functional traits

During the 2018–2020 growing season (May–September), the number of individuals of *P. australis* per plot, the number of leaves per stem, and the proportion of leaves consumed by crabs per stem were measured. The plant height was measured with a straightedge (recorded at the highest leaf height). Additionally, the length and breadth of *P. australis* leaves are also measured with a straightedge. Measurements of *P. australis* internode and spike are taken in September each year. These typical growth metrics reflect the growth of *P. australis* under nitrogen addition and crab herbivory. The intact mature leaves that were not consumed by crabs were sampled in September 2018–2020 and dried (65°C, 72 h) and ground, then sieved through 100 mesh sieves to determine the nutrient content of the leaves (C and N) by using an elemental analyzer (Vario Micro Cube, Elementar Analysensysteme GmbH, Hanau, Germany). The estimation of *P. australis* biomass at the end of each growing season (September) was conducted using regression equations, wherein stem height served as a predictor for stem biomass, aiming to prevent plant disturbance caused by biomass harvesting. The estimation equations can be found in Zhang et al. (2021), with details.

### Data analysis

The effects of nitrogen addition, crab treatment, and month (year) on soil properties and plant performances were analyzed using a linear mixed-effects model lmer() function, which is capable of handling repeated measurements. The mixed model explicitly accounted for the correlation between repeated measurements in each plot by considering nitrogen addition, crab treatment, and month (year) as fixed factors and plot as a random factor. Multiple comparisons were conducted using the ls. means () function to compare means across treatments. All data in the text are presented as mean ± SE.

We employed structural equation modeling (SEM) to estimate the direct and indirect drivers of *P. australis* growth traits. In structural equation modeling, the maximum likelihood estimation method was used to fit the model, and the optimal fitting model was selected by criterion: the *p*-value of Chi-square (Chi.sq; > 0.05), the comparative fit index (CFI; > 0.95), the standardized root mean square residual (SRMR; < 0.08), and the root mean square error of approximation (RMAEA; < 0.05).

Statistical analysis and plotting were performed using R 4.2.3 with the “lmerTest”, “ggplot2”, “lavaan”, “semPlot”, and “piecewiseSEM” packages (Cheung, 2014; Lefcheck, 2016; Wickham., 2016; Kuznetsova et al., 2017).

## Results

### The effects of nitrogen addition and crab treatment on leaf nutrients

**Figure 3:**
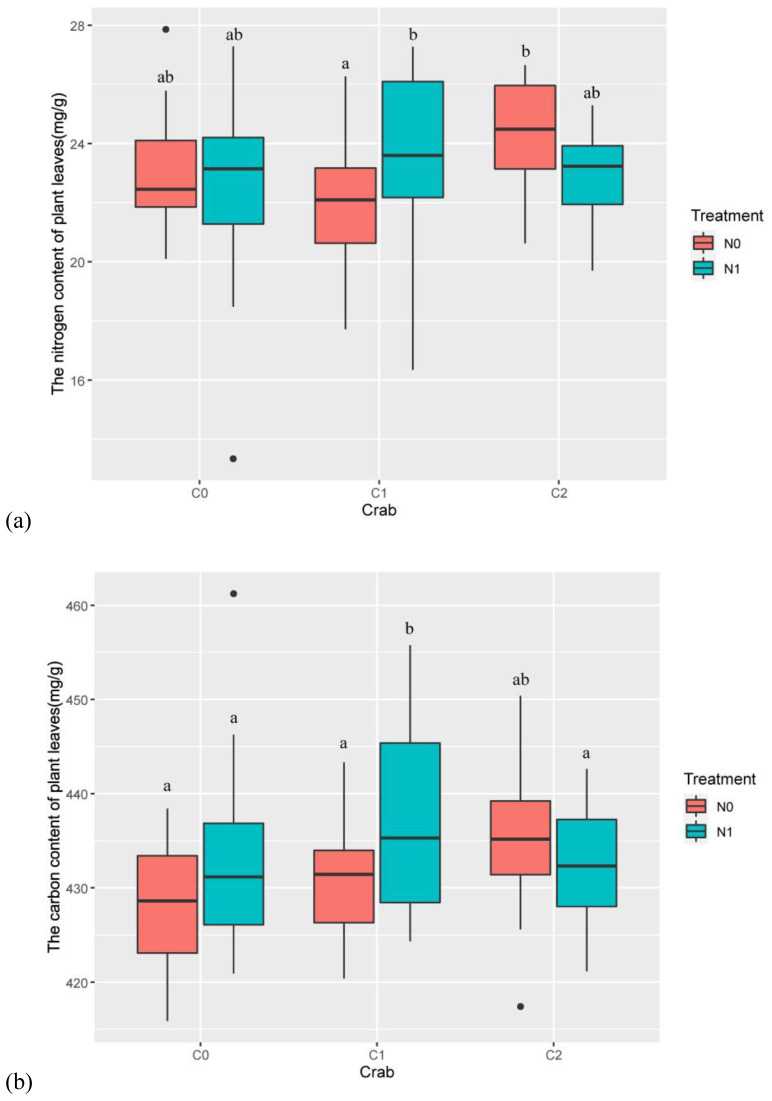
The effects of nitrogen addition and crab treatment on leaf nutrients. **(A)** The nitrogen content of *P. australis* leaves. **(B)** The carbon content of *P. australis* leaves. The red color box (N0) is without nitrogen addition, and the lake blue color box (N1) is nitrogen addition. C0 is the ambient crab treatment, C1 is the procedural crab cage treatment, and C2 is the crab exclusion cage treatment. The letters indicate the multiple comparison results; the treatments with the same letter have no significant differences.

Year (num DF = 2, den DF = 43.89, *F* = 8.75, *p*-value < 0.001), the interaction of nitrogen addition and crab treatment (num DF = 2, den DF = 23.70, *F* = 4.20, *p*-value < 0.05), and the interaction of nitrogen addition, crab treatment, and year (num DF = 4, den DF = 43.13, *F* = 2.64, *p*-value < 0.05) significantly influenced the total nitrogen content of *P. australis* leaves (**Table 1**). Nitrogen addition significantly increased the total nitrogen content of *P. australis* leaves in the C1 and had no significant differential effect on the total nitrogen content of *P. australis* leaves in the C0 and C2 (**Figure 3A**).

**Table 1:** Linear mixed-effects model predicting the influences of nitrogen addition, crab treatment, and year on the leaf carbon and nitrogen content and the biomass of *P. australis*.

Effects	Total nitrogen content *p*-value	Total carbon content *p*-value	Biomass *p*-value
Fixed effects			
Nitrogen	0.970	< 0.050^*^	0.925
Crab	0.163	0.095	< 0.001^***^
Year	< 0.001^***^	< 0.010^**^	< 0.001^***^
Nitrogen: crab	< 0.050^*^	< 0.050^*^	0.345
Nitrogen: year	0.677	0.372	0.414
Crab: year	0.139	0.232	< 0.001^***^
Nitrogen: crab: year	< 0.050^*^	< 0.050^*^	0.303

Nitrogen, crab, and year were considered fixed factors. Plot was treated as a random factor. ^***^ *p*-value < 0.001; ^**^ *p*-value < 0.01; ^*^ *p*-value < 0.05.

Nitrogen addition (num DF = 1, den DF = 26.79, *F* = 4.62, *p*-value < 0.05), year (num DF = 2, den DF = 45.42, *F* = 7.61, *p*-value < 0.01), the interaction of nitrogen addition and crab treatment (num DF = 2, den DF = 26.10, *F* = 3.88, *p*-value < 0.05), and the interaction of nitrogen addition, crab treatment, and year (num DF = 4, den DF = 44.76, *F* = 3.18, *p*-value < 0.05) significantly influenced the total carbon content of *P. australis* leaves (**Table 1**). Nitrogen addition significantly increased the total carbon content of *P. australis* leaves in the C1 and had no significant differential effect on the total carbon content of *P. australis* leaves in the C0 and C2 (**Figure 3B**).

### Effects of nitrogen addition, crab treatment, and month on consumption degree

#### Proportion of the leaf number consumed by crab in 2018

**Figure 4:**
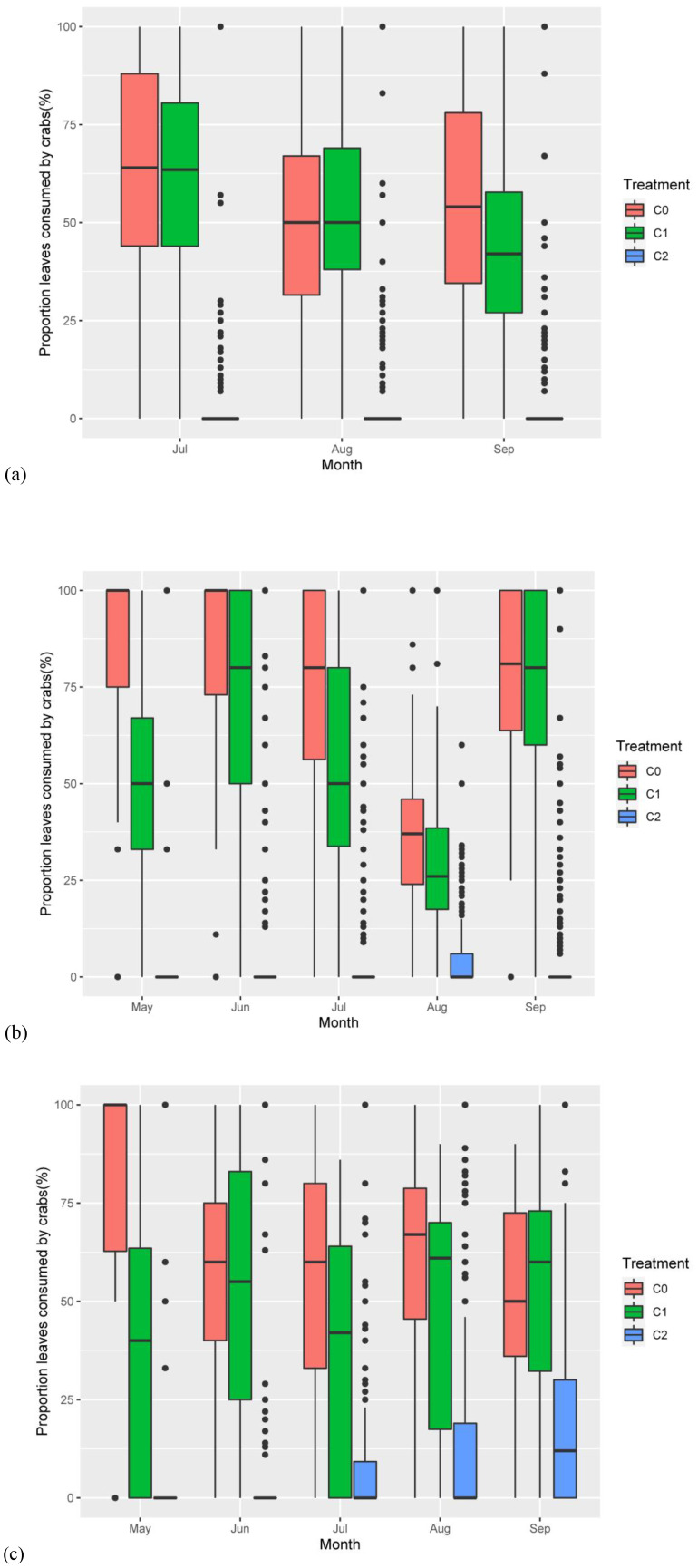
The proportion of the *P. australis* leaves consumed by crabs responding to nitrogen addition and crab treatment. **(A)** The proportion of the *P. australis* leaves consumed by crabs responding to treatments in 2018. **(B)** The proportion of the *P. australis* leaves consumed by crabs responding to treatments in 2019. **(C)** The proportion of the *P. australis* leaves consumed by crabs responding to treatments in 2020. The red color box (C0) is ambient crab treatment, the green box (C1) is procedural crab cage treatment, and the blue one (C2) is crab exclusion cage treatment.

Crab treatment (num DF = 2, den DF = 23.06, *F* = 146.47, *p*-value < 0.001), month (num DF = 2, den DF = 1,696.36, *F* = 19.06, *p*-value < 0.001), the interaction of nitrogen addition and month (num DF = 2, den DF = 1,696.36, *F* = 4.03, *p*-value < 0.05), and the interaction of crab treatment and month (num DF = 4, den DF = 1,696.37, *F* = 11.46, *p*-value < 0.001) significantly influenced the proportion of leaves per stem (**Table 2**). The result showed that C2 significantly reduced the proportion of the leaf number per stem consumed by crabs (**Figure 4A**). While 56.37% ± 1.24% and 51.73% ± 1.16% of the leaves per stem exhibited some degree of crab damage in the C0 and C1, respectively, only 3.94% ± 0.52% of the stems exhibited any evidence of grazing in the C2.

**Table 2:** Linear mixed-effects model predicting influences of nitrogen addition, crab treatment, and month on the proportion of the number of leaves consumed by crabs from 2018 to 2020.

Effects	Year 2018	Year 2019	Year 2020
	*p*-value	*p*-value	*p*-value
Fixed effects			
Nitrogen	0.364	0.989	< 0.050^*^
Crab	< 0.001^***^	< 0.001^***^	< 0.001^***^
Month	< 0.001^***^	< 0.001^***^	0.877
Nitrogen: crab	0.606	0.862	0.082
Nitrogen: month	< 0.050^*^	0.338	0.579
Crab: month	< 0.001^***^	< 0.001^***^	< 0.001^***^
Nitrogen: crab: month	0.597	0.307	< 0.098

Nitrogen, crab, and month were considered fixed factors. Plot was treated as random factors. ^***^ *p*-value < 0.001; ^**^ *p*-value < 0.01; ^*^ *p*-value < 0.05.

#### Proportion of the leaf number consumed by crab in 2019

Crab treatment (num DF = 2, den DF = 23.43, *F* = 504.72, *p*-value < 0.001), month (num DF = 4, den DF = 1,997.33, *F* = 62.367, *p*-value < 0.001), and the interaction of crab treatment and month (num DF = 8, den DF = 1,996.15, *F* = 32.94, *p*-value<0.001) significantly influenced the proportion of leaves per stem (**Table 2**). Nitrogen addition had no significant effect on the proportion of leaves that were consumed; the C2 significantly reduced the proportion of leaves that were consumed compared to the C0 and C1 (**Figure 4B**), where 71.54% ± 1.59% and (56.60% ± 1.85% of the stems were consumed by crabs in the C0 and C1, respectively, whereas only 5.78% ± 0.44% of the stems were consumed in the C2.

#### Proportion of the leaf number consumed by crab in 2020

Nitrogen addition (num DF = 1, den DF = 18.84, *F* = 4.61, *p*-value < 0.05), crab treatment (num DF = 2, den DF = 16.50, *F* = 20.43, *p*-value < 0.001), and the interaction of crab treatment and month (num DF = 8, den DF = 1,904.60, *F* = 8.59, *p*-value < 0.001) significantly influenced the proportion of leaves per stem (**Table 2**; **Figure 4C**). Similar to the results of the previous 2 years, the C2 significantly reduced the proportion of the leaf number consumed, with 57.92% ± 2.56% and (48.88% ± 3.21% of leaves per stem in the C0 and C1, respectively, and only 10.47% ± 0.47% of the stems and leaves in the C2, thus C2 successfully reduced crab consumptive impacts on *P. australis*.

### The effect of nitrogen addition, crab treatment, and month on plant performances from 2018 to 2020

#### Individuals

##### The effect of treatments on P. australis individuals in 2018

Month (num DF = 4, den DF = 96, *F* = 62.66, *p*-value < 0.001) and the interaction of crab treatment and month (num DF = 8, den DF = 96, *F* = 2.74, *p*-value < 0.01) significantly influenced the *P. australis* individuals (**Supplementary Table S1**). The results of multiple comparisons showed the *P. australis* individuals under each treatment tended to increase as the month increased; there was no significant difference in the effects of the three treatments on the *P. australis* individuals in May, June, July, and August. However, in September, the individuals were significantly higher in the C2 (25.8 ± 2.53) compared to the C0 (19.1 ± 2.49, *p*-value < 0.001) and C1 (18.2 ± 2.13, *p*-value < 0.001) (**Supplementary Figure S2**).

##### The effect of treatments on *P. australis* individuals in 2019

Crab treatment (num DF = 2, den DF = 24, *F* = 32.21, *p*-value < 0.001) and month (num DF = 4, den DF = 96, *F* = 6.56, *p*-value < 0.001) significantly influenced the *P. australis* individuals (**Supplementary Table S1**). The C0 and C1 had no significant effect on the *P. australis* individuals, whereas C2 (27.38 ± 1.47) significantly increased *P. australis* individuals compared to C0 (7.64 ± 0.57, *t*-value = − 6.63, *p*-value < 0.001) and C1 (5.82 ± 0.54, *t*-value = − 7.24, *p*-value < 0.001) (**Supplementary Figure S2**), indicating that C2 successfully reduced the effect on *P. australis* consumed by crabs.

##### The effect of treatments on *P. australis* individuals in 2020

Crab treatment (num DF = 2, den DF = 24, *F* = 66.00, *p*-value < 0.001), month (num DF = 4, den DF = 96, *F* = 55.28, *p*-value < 0.001), and the interaction of crab treatment and month (num DF = 8, den DF = 96, *F* = 46.86, *p*-value < 0.001) significantly influenced the *P. australis* individuals (**Supplementary Table S1**). During the 2020 growing season (May–September), there was an overall increasing trend in the number of *P. australis* individuals. With the individuals in the C2 being significantly higher than those in the C0 (*t*-value = − 9.76, *p*-value < 0.001) and C1 (*t*-value = − 10.13, *p*-value < 0.001), the difference becomes more and more pronounced as the year increased (**Supplementary Figure S2**).

#### Height

##### The effect of treatments on *P. australis* height in 2018

Month (num DF = 4, den DF = 2,354.45, *F* = 120.64, *p*-value < 0.001) and the interaction of crab treatment and month (num DF = 8, den DF = 2,354.48, *F* = 2.01, *p*-value < 0.05) significantly influenced the *P. australis* height (**Table 3**). There was no significant effect on *P. australis* height under the three treatments in May, June, and July, but it was significantly higher under the C2 than C0 in August (78.92 cm ± 1.54 cm vs. 72.33 cm ± 1.91 cm, *t*-value = − 2.03, *p*-value < 0.05) and September (86.61 cm ± 1.57 cm vs. 78.63 cm ± 1.26 cm, *t*-value = −2.60, *p*-value < 0.05). The interaction of nitrogen addition and crab treatment had no significant effect on *P. australis* height (**Supplementary Figure S3**).

**Table 3:** Linear mixed-effects model predicting influences of the nitrogen addition, crab treatment, and month on *P. australis* growth indicators from 2018 to 2020.

Effects	Year 2018			Year 2019			Year 2020		
	Height	Leaf length	Leaf breadth	Height	Leaf length	Leaf breadth	Height	Leaf length	Leaf breadth
Fixed effects									
Nitrogen	0.164	0.481	0.688	0.811	0.399	0.367	< 0.050^*^	< 0.050^*^	< 0.050^*^
Crab	0.722	0.861	0.396	< 0.001^***^	< 0.001^***^	0.193	< 0.001^***^	< 0.010^**^	< 0.050^*^
Month	< 0.001^***^	< 0.001^***^	< 0.010^**^	< 0.001^***^	< 0.001^***^	< 0.001^***^	< 0.001^***^	< 0.010^**^	< 0.001^***^
Nitrogen: crab	0.875	0.725	0.792	0.142	0.530	0.731	< 0.050^*^	< 0.050^*^	< 0.050^*^
Nitrogen: month	0.848	0.122	0.289	0.475	0.067	0.360	0.984	0.698	0.733
Crab: month	< 0.050^*^	0.713	0.184	0.946	< 0.001^***^	0.063	0.974	< 0.010^**^	< 0.001^***^
Nitrogen: crab: month	0.278	0.254	0.529	0.495	0.628	0.381	0.615	0.684	0.343

Nitrogen, crab, and month were considered fixed factors. Plot was treated as a random factor. ^***^ *p*-value < 0.001; ^**^ *p*-value < 0.01; ^*^ *p*-value < 0.05.

##### The effect of treatments on *P. australis* height in 2019

Crab treatment (num DF = 2, den DF = 22.89, *F* = 29.89, *p*-value < 0.001), month (num DF = 4, den DF = 1,999.02, *F* = 67.97, *p*-value < 0.001) significantly influenced the *P. australis* height (**Table 3**). The effect on *P. australis* height varied significantly between months; it was significantly higher in September than in other months and significantly higher in the C2 (68.9 cm ± 0.58 cm) than in the C0 (54.02 cm ± 1.09 cm, *t*-value = − 7.58, *p*-value < 0.001) and C1 (61.19 cm ± 1.30 cm, *t*-value = − 4.27, *p*-value < 0.001), and the plant height of C1 was significantly higher than C0 (*t*-value = − 2.55, *p*-value < 0.05) (**Supplementary Figure S3**).

##### The effect of treatments on *P. australis* height in 2020

**Figure 5:**
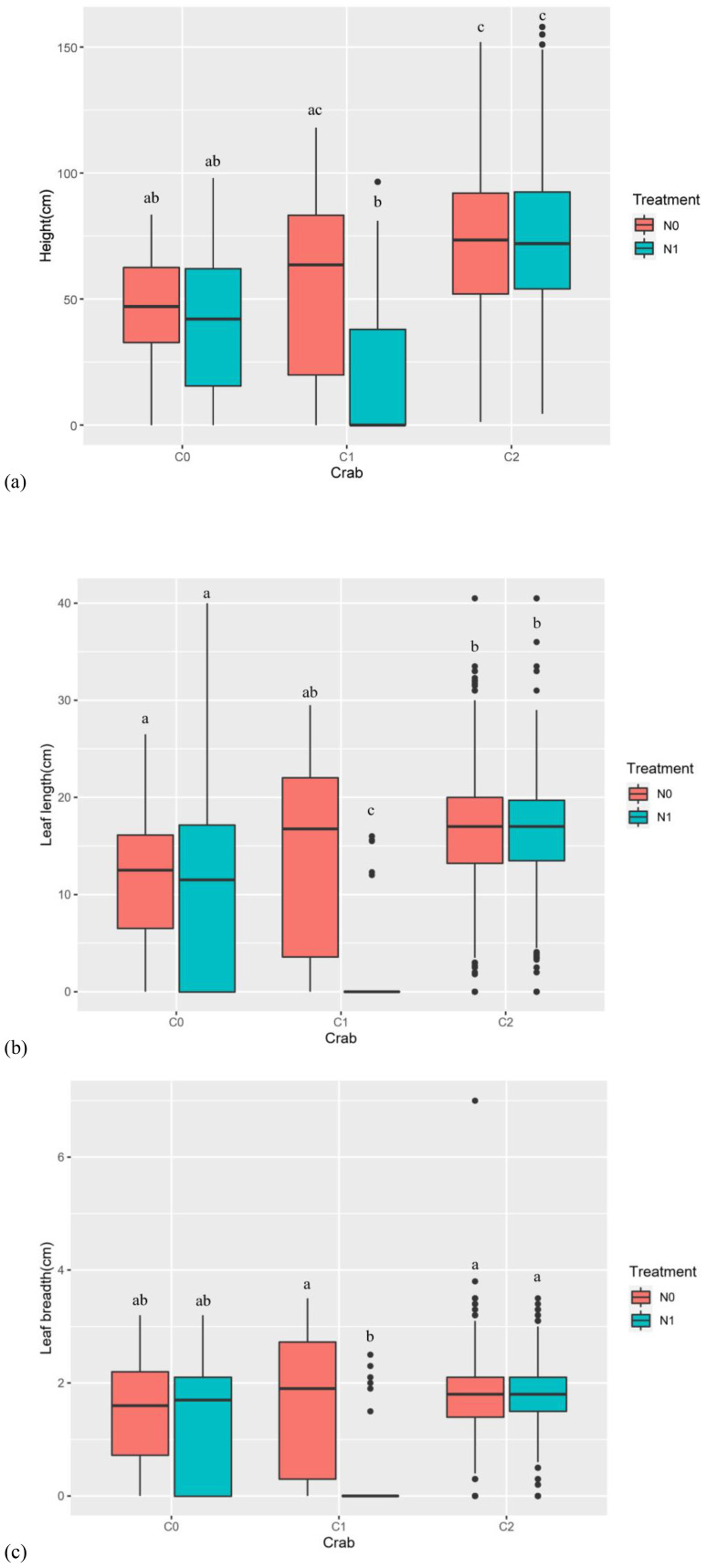
The effect of nitrogen addition and crab treatment on *P. australis* height, leaf length, and breadth in 2020. **(A)** The effect of treatments on the *P. australis* height in 2020. **(B)** The effect of treatments on the *P. australis* leaf length in 2020. **(C)** The effect of treatments on the *P. australis* leaf breadth in 2020. The red color box (N0) is without nitrogen addition, the lake blue color box (N1) is nitrogen addition. C0 is the ambient crab treatment, C1 is the procedural crab cage treatment, and C2 is the crab exclusion cage treatment. The letters indicate the multiple comparison results; the treatments with the same letter have no significant differences.

Nitrogen addition (num DF = 1, den DF = 17.13, *F* = 5.06, *p*-value < 0.05), crab treatment (num DF = 2, den DF = 17.18, *F* = 14.15, *p*-value < 0.001), month (num DF = 4, den DF = 1,909.75, *F* = 12.18, *p*-value < 0.001), and the interaction of nitrogen addition and crab treatment (num DF = 2, den DF = 17.18, *F* = 4.17, *p*-value < 0.05) significantly influenced the *P. australis* height (**Table 3**). When nitrogen was applied, the C2 significantly increased plant height compared to the C0 and C1, while there was no significant effect on plant height between the other two treatments, indicating that crab consumption was the key factor contributing to the effect on plant height. Moreover, nitrogen addition significantly reduced *P. australis* height in the C1, while having no significant effect in the C0 and C2 (**Figure 5A**).

#### Leaf number, leaf length, and breadth

##### The effect of treatments on leaf number from 2018 to 2020

Month (num DF = 4, den DF = 2,355.96, *F* = 284.97, *p*-value < 0.001), the interaction of crab treatment and month (num DF = 8, den DF = 2,355.98, *F* = 4.82, *p*-value < 0.001), the interaction of nitrogen addition, crab treatment, and month (num DF = 8, den DF = 2,355.98, *F* = 2.10, *p*-value < 0.05) significantly influenced the leaf number (**Supplementary Table S1**). Leaf numbers varied significantly between months, with a general trend of increasing leaf numbers with increasing months in 2018 (**Supplementary Figure S4**).

Crab treatment (num DF = 2, den DF = 22.60, *F* = 34.28, *p*-value < 0.001) and month (num DF = 4, den DF = 1,997.10, *F* = 138.90, *p*-value < 0.001) significantly influenced the leaf number in 2019 (**Supplementary Table S1**). There was a significant difference in the leaf number between months, with significantly more leaves in September than in other months (**Supplementary Figure S4**). The leaf number in the C2 (6.86 ± 0.09) was significantly higher than that in the C0 (4.77 ± 0.17, *t*-value = − 8.07, *p*-value < 0.001) and C1 (5.73 ± 0.23, *t*-value = − 4.69, *p*-value < 0.001). Moreover, the leaf number in the C1 was significantly higher than that in the C0 (*t*-value = − 2.51, *p*-value < 0.05), indicating that crab consumption had an effect on the leaf number.

Crab treatment (num DF = 2, den DF = 13.43, *F* = 20.77, *p*-value < 0.001) and month (num DF = 4, den DF = 1,905.63, *F* = 24.05, *p*-value < 0.001) significantly influenced the leaf number in 2020 (**Supplementary Table S1**). Leaf number differed significantly among months except for July and August (**Supplementary Figure S4**), and the C2 (7.62 ± 0.10) significantly increased the *P. australis* leaf number compared to the C0 (4.72 ± 0.31, *t*-value = − 4.83, *p*-value < 0.001) and C1 (4.38 ± 0.33, *t*-value = − 5.58, *p*-value < 0.001). The C0 and C1 had no significant effect on leaf number.

##### The effect of treatments on leaf length from 2018 to 2020

Month (num DF = 4, den DF = 2,350.55, *F* = 10.89, *p*-value < 0.001) significantly influenced the leaf length in 2018 (**Table 3**). Crab treatment (num DF = 2, den DF = 21.32, *F* = 10.74, *p*-value < 0.001), month (num DF = 4, den DF = 1,993.15, *F* = 6.82, *p*-value < 0.001), and the interaction of crab treatment and month (num DF = 8, den DF = 1,991.50, *F* = 6.52, *p*-value < 0.001) significantly influenced the leaf length in 2019 (**Table 3**); compared to the C0, the C2 significantly increased leaf length. The interaction of nitrogen addition and crab treatment had no significant effect on leaf length in 2018 and 2019 (**Supplementary Figure S3**).

In 2020, nitrogen addition (num DF = 1, den DF = 20.32, *F* = 5.32, *p*-value < 0.05), crab treatment (num DF = 2, den DF = 20.02, *F* = 9.39, *p*-value < 0.01), month (num DF = 4, den DF = 1,912.27, *F* = 4.16, *p*-value < 0.01), the interaction of nitrogen addition and crab treatment (num DF = 2, den DF = 20.02, *F* = 5.02, *p*-value < 0.05), and the interaction of crab treatment and month (num DF = 8, den DF = 1,912.74, *F* = 3.12, *p*-value < 0.01) significantly influenced the leaf length (**Table 3**). Moreover, nitrogen addition significantly reduced the leaf length in C1, whereas nitrogen addition had no significant effect on the leaf length in C0 and C2 (**Figure 5B**).

##### The effect of treatments on leaf breadth from 2018 to 2020

Month significantly influenced the leaf breadth in 2018 (num DF = 4, den DF = 2,350.18, *F* = 3.47, *p*-value < 0.01) and 2019 (num DF = 4, den DF = 1,992.83, *F* = 8.99, *p*-value < 0.001) (**Table 3**). The interaction of nitrogen addition and crab treatment have no significant effect on leaf breadth in 2018 and 2019 (**Supplementary Figure S3**). In 2019, leaf breadth tended to increase significantly between May and July; there was no significant difference in the leaf breadth between July and September.

In 2020, nitrogen addition (num DF = 1, den DF = 21.38, *F* = 4.68, *p*-value < 0.05), crab treatment (num DF = 2, den DF = 21.27, *F* = 3.66, *p*-value < 0.05), month (num DF = 4, den DF = 1,912.33, *F* = 6.08, *p*-value < 0.001), the interaction of nitrogen addition and crab treatment (num DF = 2, den DF = 21.27, *F* = 3.80, *p*-value < 0.05), and the interaction of crab treatment and month (num DF = 8, den DF = 1,912.60, *F* = 4.86, *p*-value < 0.001) significantly influenced the leaf breadth (**Table 3**). Moreover, nitrogen addition significantly reduced the *P. australis* leaf breadth in the C1, whereas nitrogen addition had no significant effect on the *P. australis* leaf breadth in the C0 and C2 (**Figure 5C**).

#### Internode and spike

##### The effect of treatments on internode length from 2018 to 2020

Nitrogen addition and crab treatment and their interaction had no significant effect on the *P. australis* internode length in 2018. Crab treatment (num DF = 2, den DF = 35.07, *F* = 25.78, *p*-value < 0.001) significantly influenced the *P. australis* internode length in 2019. The C2 (6.29 cm ± 0.11 cm) significantly increased the *P. australis* internode compared to the C0 (4.40 cm ± 0.20 cm) and C1 (4.35 cm ± 0.21 cm) (**Supplementary Figure S5**). In 2020, crab treatment (num DF = 2, den DF = 85.73, *F* = 29.52, *p*-value < 0.001) also significantly influenced the *P. australis* internode length (**Supplementary Table S1**). The C2 significantly increased the *P. australis* internode length compared to the C0 and C1 (**Supplementary Figure S5**). This suggests that crab herbivory significantly affects *P. australis* internode.

##### The effect of treatments on spike length from 2018 to 2020

Crab Treatment (num DF = 2, den DF = 23.25, *F* = 5.63, *p*-value < 0.05) significantly influenced the *P. australis* spike length in 2018. The C2 (2.14 cm ± 0.28 cm) significantly increased spike length compared to the C0 (0.24 cm ± 0.10 cm) and C1 (1.03 cm ± 0.17 cm). Nitrogen addition and crab treatment and their interaction had no significant effect on the *P. australis* spike in 2019 and 2020 (**Supplementary Table S1**).

#### Biomass

To avoid damage to the plots, biomass was measured in September 2020, and the biomass from 2018 to 2020 was estimated according to the relationship between plant height and biomass. The results showed that the biomass estimated in 2020 was linearly related to the actually measured biomass (**Supplementary Figure S6**, *R* ^2^ = 0.99), thus the biomass estimation data could be used for analysis.

**Figure 6:**
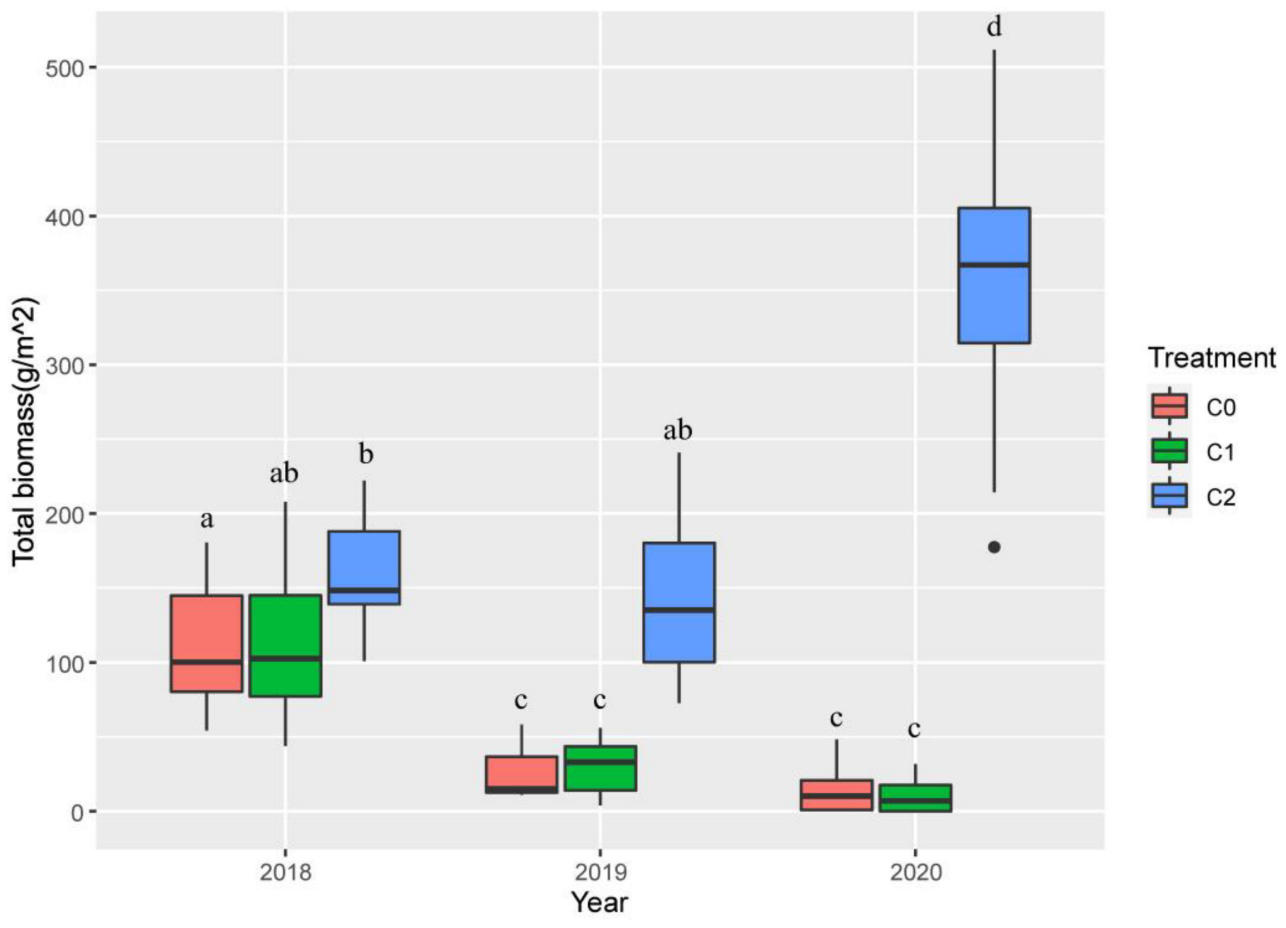
The effect of nitrogen addition, crab treatment, and year on *P. australis* biomass from 2018 to 2020. The red color box (C0) is ambient crab treatment, the green box (C1) is procedural crab cage treatment, and the blue one (C2) is crab exclusion cage treatment. The letters in (a–c) indicate the multiple comparison results; the treatments with the same letter have no significant differences.

Crab treatment (num DF = 2, den DF = 24, *F* = 60.48, *p*-value < 0.001), year (num DF = 2, den DF = 48, *F* = 31.97, *p*-value < 0.001) and the interaction of crab treatment and year (num DF = 4, den DF = 48, *F* = 66.53, *p*-value < 0.001) significantly influenced the total biomass of plant (**Table 1**). In 2018, C2 significantly increased plant biomass (159.86 g/m^2^ ± 13.04 g/m^2^, *t*-value = − 2.25, *p*-value < 0.05) compared to C0 (110.93 g/m^2^ ± 13.83 g/m^2^), and in 2019 and 2020, compared to the other two treatments, plant biomass of C2 reached the highest value in 2020 (354.10 g/m^2^ ± 32.16 g/m^2^) (**Figure 6**).

### The effect of nitrogen addition, crab treatment, and year on environmental factors from 2018 to 2020

#### Soil pH

Crab treatment (num DF = 2, den DF = 24, *F* = 14.31, *p*-value < 0.001), year (num DF = 2, den DF = 48, *F* = 20.48, *p*-value < 0.001), the interaction of crab treatment and year (num DF = 4, den DF = 48, *F* = 14.16, *p*-value < 0.001), and the interaction of crab treatment, nitrogen addition, and year (num DF = 4, den DF = 48, *F* = 5.36, *p*-value < 0.01) significantly influenced soil pH (**Supplementary Table S2**). Soil pH of N0C2 in 2020 was the lowest (8.48 ± 0.08), compared to 2018 and 2019. N0C0, N0C1, N1C0, N1C1, and N1C2 significantly increased soil pH in 2020; furthermore, there were no significant differences in soil pH between the treatments in 2018 and 2019 (**Supplementary Figure S7**).

#### Soil electronic conductivity

Crab treatment (num DF = 2, den DF = 24, *F* = 6.40, *p*-value < 0.01), year (num DF = 2, den DF = 48, *F* = 1,129.32, *p*-value < 0.001), and the interaction of crab treatment and year (num DF = 4, den DF = 48, *F* = 9.70, *p*-value < 0.001) significantly influenced the soil electronic conductivity (**Supplementary Table S2**). The soil electronic conductivity exhibited a pattern of initial increase followed by a decrease between 2018 and 2020, with significantly lower soil electronic conductivity observed in 2020 under the treatments of C0, C1, and C2 compared to 2018 and 2019 (**Supplementary Figure S7**).

#### The total nitrogen content of the soil

Year (num DF = 2, den DF = 47.60, *F* = 6.80, *p*-value < 0.01) and the interaction of nitrogen addition, crab treatment, and year (num DF = 4, den DF = 47.59, *F* = 2.67, *p*-value < 0.05) significantly influenced the total nitrogen content of soil (**Supplementary Table S2**). There was no significant effect of treatment groups on the total nitrogen content of the soil in 2018. Compared to the N0C0, the N1C0 and the N1C1 in 2019 significantly reduced the total nitrogen content of the soil (*p*-value < 0.01), and the total nitrogen content of the soil was highest under the N0C0 (0.68 mg/g ± 0.03 mg/g). In 2020, the total nitrogen content of the soil was significantly increased under the N0C2 and reached the maximum value in this year (0.60 mg/g ± 0.04 mg/g), while the other treatments had no significant effect on the total nitrogen content of the soil (**Supplementary Figure S7**).

### Direct and indirect drivers of *P. australis* functional traits

**Figure 7:**
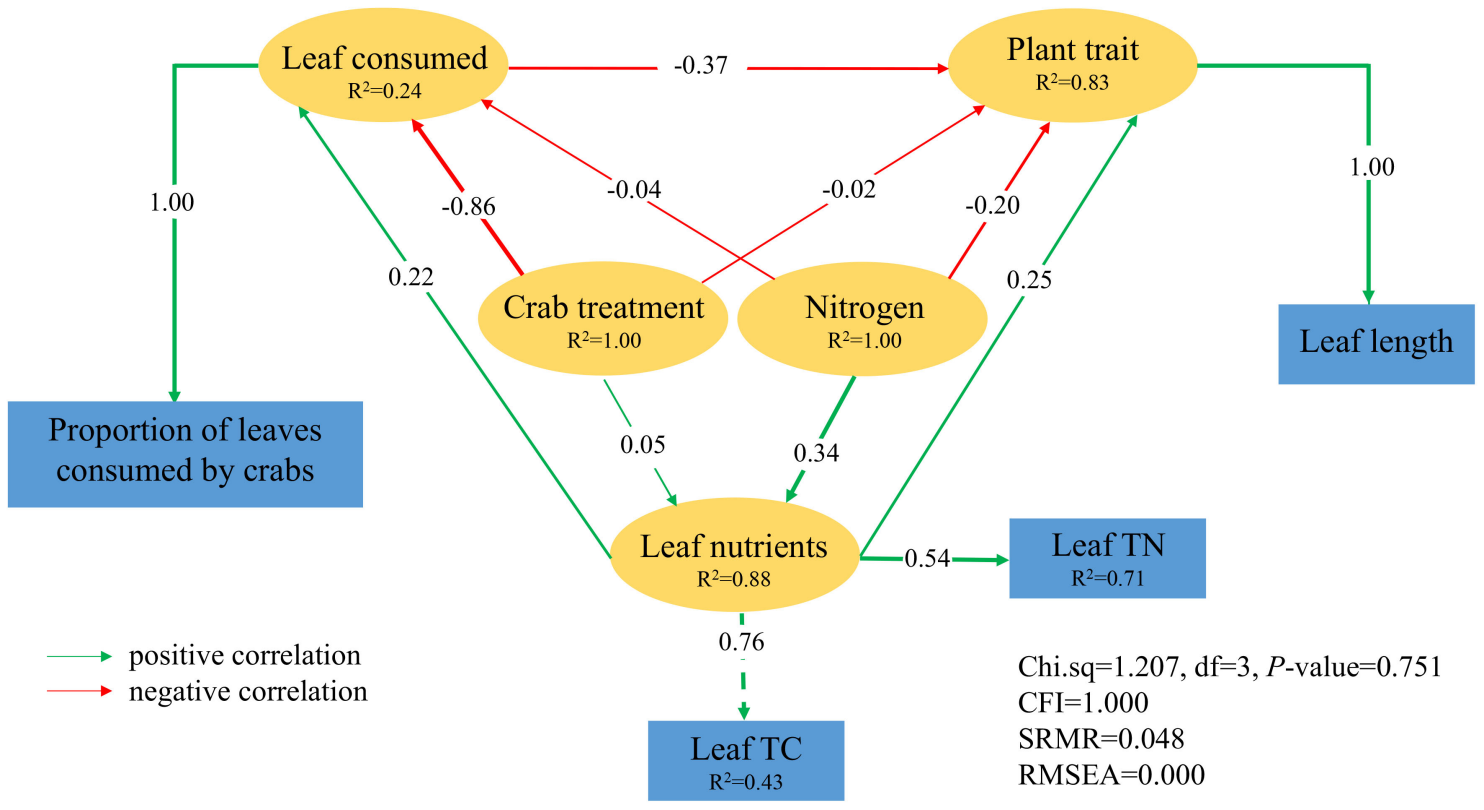
The result of structural equation modeling on the consequences of nitrogen addition and crab treatment in 2018. The green line indicates a positive correlation, the red line indicates a negative correlation, the solid line indicates direct influences, the width of the line and arrow indicates the strength of the relationship, and *R* ^2^ indicates the proportion of the total variance explained by the model.

The structural equation models, based on data from 2018, 2019, and 2020, demonstrated an excellent fit. Nitrogen addition exhibited a direct positive impact on leaf nutrients in 2018 (path coefficient = 0.34), and leaf nutrients had a positive direct effect on the proportion of leaves consumed by crabs (path coefficient = 0.22). In contrast, crab treatment had a direct negative effect on the proportion of leaves consumed by crabs (path coefficient = − 0.86), indicating that excluding crabs resulted in decreased leaf consumption by them. Furthermore, *P. australis* functional traits were adversely affected by the proportion of leaves consumed by crabs (path coefficient = − 0.37), suggesting reductions in plant traits such as leaf length (**Figure 7**).

The structural equation modeling analysis conducted using data from 2019 revealed a positive direct effect of nitrogen addition on leaf nutrients (path coefficient = 0.11). Additionally, leaf nutrients exhibited a positive direct effect on the proportion of leaves consumed by crabs (path coefficient = 0.08). In contrast, crab treatment had a direct negative effect on the proportion of leaves consumed by crabs (path coefficient = − 0.86), consistent with the findings from 2018. Furthermore, the proportion of leaves consumed by crabs negatively impacted *P. australis* functional traits (biomass and individuals; path coefficient = − 0.51) (**Supplementary Figure S8A**). Additionally, leaf nutrients exhibited a direct negative impact on plant functional traits (path coefficient = − 0.49), suggesting that nitrogen addition indirectly and positively influenced the plant traits in 2019 by altering leaf nutrients rather than through crab consumption.

Model results from 2020 indicate that, in contrast to the years 2018 and 2019, nitrogen addition had a negative impact on leaf nutrients (path coefficient = − 0.22) and the proportion of leaves consumed (path coefficient = − 0.16). Furthermore, it was observed that the proportion of leaves consumed by crabs negatively affected plant growth traits represented by individuals (path coefficient = − 0.33; **Supplementary Figure S8A**). Additionally, nitrogen addition resulted in a direct decrease in the proportion of leaves consumed by crabs (path coefficient = − 0.30; **Supplementary Figure S8A**). Moreover, nitrogen addition directly decreased the proportion of leaves consumed by crabs, thereby indirectly enhancing plant traits (path coefficient = 0.10). Conversely, crab treatment directly influenced the proportion of leaves consumed by crabs in a negative manner (path coefficient = − 0.50), consistent with the findings from 2018 and 2019 (**Supplementary Figure S8A**).

## Discussion

### The effect of a bottom–up factor on the plant growth in the salt marsh

It is generally acknowledged that the control of plant communities by the bottom-up effect is undoubtedly pervasive since herbivorous animals as well as other consumers could not exist without plants (Holdredge et al., 2008). Salinity, tides, and nutrients are generally recognized as the most important bottom–up factors affecting salt marsh plant communities (Nystrom et al., 2003). Among these bottom–up factors, nitrogen is an essential element that plays an important role in plant growth and is regarded as one of the crucial limiting factors (Weber and Burow, 2018). Our findings demonstrated that nitrogen addition significantly increased the total nitrogen and total carbon content in *P. australis* leaves. Furthermore, in 2020, nitrogen addition not only significantly increased plant height but also increased leaf length and leaf breadth of *P. australis.* These results are consistent with previous studies indicating elevated levels of nitrogen deposition, increased nutrient availability, and altered plant functional traits (Wang et al., 2017; Guan et al., 2019; Zhang et al., 2022).

There were significant variations in environmental conditions observed in the YRD over the 3-year study periods, with the highest values of soil electronic conductivity in 2019. Additionally, our previous study has found a decrease in both frequency and duration of flooding during spring 2019 compared to spring 2018, exacerbating the severity of drought conditions experienced that year (Zhang et al., 2021). The occurrence of spring drought can increase salinity stress, resulting in a significantly higher soil electronic conductivity in 2019 compared to both 2018 and 2020. The growth of plants is typically inhibited by elevated salinity. The detrimental effects of salinity on plant growth are generally attributed to several reasons: the direct toxicological impact of sodium and chloride ions, interference with the uptake of essential nutrients, and effects resulting from a decrease in external water potential (Nystrom et al., 2003). In addition, the proportion of *P. australis* leaves consumed by crabs in N0C1 as well as N0C2 was highest in 2019. This finding suggests that spring drought and higher salinity in 2019 inhibited the fitness of *P. australis*, particularly in the experimental plot where this dominant plant was exposed to crab herbivory. The findings of our study align with the outcomes of our previous investigation (Zhang et al., 2021) as well as those reported by other researchers. It was reported that drought triggers peaks in consumer herbivory by increasing salinity stress, leading to severe wetland vegetation mortality (He and Silliman, 2016). Similarly, Silliman et al. (2005) revealed that in the presence of drought conditions, snail herbivory resulted in extensive mortality of salt marsh vegetation in the southeastern and Gulf coastal zones of North America, suggesting the vulnerability of wetland vegetation to drought. Therefore, the availability of freshwater is crucial for the conservation of coastal wetland vegetation.

Additionally, from 2018 to 2020, the number of *P. australis* individuals showed a consistent increasing trend over time in the N0C1. Conversely, there was an overall decreasing trend observed with increasing years among the other treatments. Additionally, stem height and leaf number showed an increasing trend with increasing years in the N1C2, while there was a decreasing trend with decreasing years among the other treatments. Moreover, leaf length and internode length showed a general decreasing trend with increasing years among the N0C0, N0C1, N1C0, and N1C1; leaf breadth showed a general decreasing trend with increasing years among the N0C0, N0C1, N1C0, N1C1, and N1C2; however, spike length showed an increasing trend with increasing years among the N0C0, N0C1, N1C0, and N1C2. The findings suggest that different traits exhibit varying sensitivities to environmental changes. It is important to note that not all *P. australis* traits will be equally affected by nitrogen addition and crab herbivory. Therefore, when investigating the bottom–up and top–down effects, it is advisable to measure multiple traits (Morecroft et al., 1994; Yang et al., 2022).

### The effect of a top–down factor on the plant growth in the salt marsh

Recent studies have found that organisms occupying higher trophic levels can have a critical impact on the growth and distribution patterns of salt marsh plants through trophic cascades, either via direct consumption by herbivorous animals or indirectly by modulating the abundance of herbivory animals (Holdredge et al., 2008; Bertness et al., 2009). We found that the proportion of *P. australis* leaves consumed by crabs was significantly lower in the crab exclusion cage treatment, highlighting that crab herbivory is a nonnegligible factor affecting salt marsh vegetation. In terms of plant functional traits of *P. australis*, the crab exclusion cage treatment significantly enhanced the abundance, height, leaf number, and biomass of *P. australis*. This suggests that crab herbivory has a negative impact on the functional traits of plants. This finding is consistent with the results of previous studies. The degradation of the *Spartina* plant in New England salt marshes in the USA was attributed to crab herbivory (Keddy, 2002). Silliman and Zieman (2001) conducted an experiment using netting to establish plots with and without snail removal and found that after a 3-month period, all the mutualistic grasses in the snail-removed plots remained intact, while those in the control plots experienced complete mortality. Subsequent studies reported that crab herbivory also has a very important role in regulating the growth and distribution of salt marsh plants (Holdredge et al., 2008; Bertness et al., 2009).

The impacts of small herbivores such as crabs, snails, and insects on coastal wetland vegetation are widespread and generalized. Crab herbivory has been found to significantly regulate wetland plant performance not only in North America but also in South America (Finke and Denno, 2004; Alberti et al., 2008; Johnson and Jessen, 2008; Daleo et al., 2009). Additionally, there is compelling evidence indicating that crabs have strong control over aboveground production in salt marshes at large spatial scales in Argentina (Sala et al., 2008). The findings of our study contribute to the growing body of evidence supporting the top–down impact of crab herbivory in Asian salt marsh ecosystems. The global prevalence of strong consumer control of salt marshes implies the need for increased attention to the top–down effect on wetland vegetation.

### The interactive effect of bottom–up factor and top–down factor on plant growth

The strength of the top–down effect on the plant community is influenced by the bottom–up effect by directly affecting herbivory abundance, indirectly impacting the nutrient content of plants, or altering the herbivory activity (Bertness et al., 2008; Alberti et al., 2010). We detected the positive effect of nitrogen addition on the total nitrogen and carbon content of *P. australis* leaves in the procedural crab cage treatment, although in both the ambient crab and crab exclusion cage treatment, nitrogen addition had no such effect. The possible explanation for these results may be as follows: nitrogen addition in the ambient crab treatment resulted in a reduction of the carbon-to-nitrogen ratio of the leaves, resulting in improved palatability of the leaves (Gruner et al., 2008), and it is possible that crabs selectively fed on the stems of leaves with higher nutrient content while leaving behind those with lower nutrient content. The *P. australis* in the crab exclusion cage treatment, without the consumption by crabs, may have utilized the excess nutrients to support their own growth at a high rate following nitrogen addition, which led to the lower leaf nutrient content.

Nitrogen additions significantly increased the proportion of leaves consumed by crabs, suggesting that increased nitrogen content improved the quality of the *P. australis* leaves when nutrients were added. Moreover, the increased nitrogen content enhanced the palatability of the *P. australis* leaves, making them more susceptible to crab herbivory (Silliman and Zieman, 2001). The studies conducted in New England wetlands have shown that vegetation with higher nitrogen levels exhibited higher consumer herbivory compared to vegetation with lower nitrogen levels (Bertness et al., 2008). Additionally, investigations carried out in Southwest Atlantic wetlands have revealed that nutrient addition increased the biomass of wetland vegetation, while the presence of consumers decreased it (Daleo et al., 2015), thereby indicating an interactive effect between nutrient addition and consumers on wetland vegetation.

In 2018 and 2019, the structural equation modeling revealed a positive correlation among nitrogen addition, crab treatment, and leaf nutrient levels, indicating that these treatments enhanced the content of leaf nutrients. Conversely, we observed a negative correlation between leaf nutrient content and plant growth traits in *P. australis*, suggesting that higher leaf nutrient content was associated with reduced biomass, leaf number, and leaf length. It is confirmed that the top–down effect can be a crucial factor contributing to the degradation of the vegetation in coastal wetlands, particularly in the context of global changes such as nutrient enrichment and extreme drought, which may enhance the top–down effect. However, according to the results of structural equation modeling, the interactive effect between nitrogen addition and crab herbivory in 2020 differs from that observed in 2018 and 2019. In those 2 years, nitrogen addition increased leaf nutrient levels (TC and TN), subsequently leading to an increase in the proportion of leaves consumed by crabs, thereby negatively impacting plant functional traits. Conversely, in 2020, nitrogen addition was found to decrease leaf nutrient levels and reduce the proportion of leaves consumed by crabs, thereby promoting the plant trait. Additionally, nitrogen addition directly decreased the proportion of leaves consumed by crabs, indirectly resulting in an increase in plant traits. These divergent findings may be attributed to plant mortality occurring within several plots subjected to nitrogen addition and crab treatment (N1C1 and N1C0), where measurements for leaf nutrients and crab consumption were not feasible. This discrepancy could potentially account for the observed differences; however, it is plausible that the combined effects of nitrogen addition and crab herbivory contributed to the plant mortality observed in these plots, thereby indicating the detrimental impact of both factors on plant growth. In this regard, the synergistic effect of nitrogen addition and crab herbivory remains consistent across 3 years. Therefore, we suggested that enhancing bird predation on crabs to alleviate plant consumption stress could potentially serve as an effective strategy for restoring degraded wetland vegetation (Zhang et al., 2021; Xu et al., 2023).

## Conclusion

In summary, we conducted a 3-year field experiment manipulating nitrogen addition and crab herbivory in tidal wetlands within the Yellow River Delta. The result revealed that nitrogen addition significantly enhanced the total nitrogen and total carbon content of *P. australis* leaves. Furthermore, leaf nutrients significantly increased the proportion of leaves consumed in plots where crabs moved freely, thereby exerting a suppressing effect on the *P. australis* growth. Consequently, nutrient enrichment and crab herbivory played an important role in regulating *P. australis* growth in the tidal marshes of YRD. Therefore, our study facilitates our comprehension of the underlying factors contributing to vegetation degradation in coastal wetlands and establishes a crucial theoretical foundation for the conservation of these ecosystems in the context of global environmental changes. In the future, firstly, it is imperative to conduct comprehensive testing of nutrient enrichment and top–down effects across a diverse range of national ecosystems to obtain universally applicable results. Secondly, the investigation of the influence of plant functional traits on crab-related traits presents an intriguing avenue for research. Thirdly, there is a need for further investigation into nutrient enrichment and trophic cascades, such as incorporating higher trophic levels like avian species into the experiments.

## Data Availability

The datasets provided in this study can be accessed in the **Supplementary Material Data Sheet 2**.
