# Supplementary material for: Nitrogen enrichment enhances the negative top–down effect on plant functional traits
Source: Front Plant Sci. 2024 Aug 30;15:1418724. doi: 10.3389/fpls.2024.1418724 (PMC11397305; doi:10.3389/fpls.2024.1418724)
Supplement: Supplementary file 1 [file DataSheet1.docx]

**Supporting information**

**Table S1** Linear mixed-effects model predicting influences of the Nitrogen Addition, Crab Treatment and Month on *P. australis* growth indicators from 2018 to 2020.

| Effects | Year 2018 | | |  | Year 2019 | | |  | Year 2020 | | |  |
| --- | --- | --- | --- | --- | --- | --- | --- | --- | --- | --- | --- | --- |
|  | Individuals | Leaf number | Internode | Spike | Individuals | Leaf number | Internode | Spike | Individuals | Leaf number | Internode | Soike |
| Fixed effects |  |  |  |  |  |  |  |  |  |  |  |  |
| Nitrogen | 0.961 | 0.952 | 0.283 | 0.124 | 0.721 | 0.407 | 0.342 | 0.516 | 0.996 | 0.124 | 0.065 | 0.203 |
| Crab | 0.330 | 0.094 | 0.798 | <0.050* | <0.001  *** | <0.001  *** | <0.001  *** | 0.598 | <0.001*** | <0.001*** | <0.001  *** | 0.481 |
| Month | <0.001*** | <0.001*** | —— | —— | <0.001  *** | <0.001  *** | —— | —— | <0.001*** | <0.001*** | —— | —— |
| Nitrogen: Crab | 0.437 | 0.593 | 0.197 | 0.525 | 0.308 | 0.336 | 0.325 | 0.598 | 0.729 | <0.056 | 0.167 | 0.372 |
| Nitrogen: Month | 0.995 | 0.962 | —— | —— | 0.831 | 0.961 | —— | —— | 0.999 | 0.961 | —— | —— |
| Crab: Month | <0.010** | <0.001** | —— | —— | 0.247 | 0.448 | —— | —— | <0.001*** | <0.894 | —— | —— |
| Nitrogen: Crab: Month | 0.947 | <0.050* | —— | —— | 0.260 | 0.416 | —— | —— | 0.990 | 0.644 | —— | —— |

Note: Nitrogen addition, Crab and Month were considered fixed factors; plot was treated as random factors. ‘***’ indicates *P*-value < 0.001; ‘**’ indicates *P*-value < 0.01; ‘*’ indicates *P*-value < 0.05; ——indicates no data.

**Table S2** Linear mixed-effects model predicting influences of Nitrogen Addition, Crab Treatment and Year on the environmental factor

| Effects | Soil pH | Soil electronic conductivity | Total nitrogen  content of soil |
| --- | --- | --- | --- |
| Fixed effects |  |  |  |
| Nitrogen | 0.628 | 0.423 | 0.113 |
| Crab | <0.001^***^ | <0.010^**^ | 0.085 |
| Year | <0.001^***^ | <0.001^***^ | <0.010^**^ |
| Nitrogen: Crab | 0.709 | 0.578 | 0.612 |
| Nitrogen: Year | 0.149 | 0.429 | 0.119 |
| Crab: Year | <0.001^***^ | <0.001^***^ | 0.799 |
| Nitrogen: Crab: Year | <0.010^**^ | 0.176 | <0.050^*^ |

Note: Nitrogen addition, Crab and Year were considered fixed factors; plot was treated as random factors. ‘***’ indicates *P*-value < 0.001; ‘**’ denotes *P*-value < 0.01; ‘*’ denotes *P*-value < 0.05.

**Figure S1** Experimental setup diagram. (a) The Ambient Crab Treatment (C0); (b) The Procedural Crab Cage Treatment (C1); (c) The Crab Exclusion Cage Treatment (C2).

**
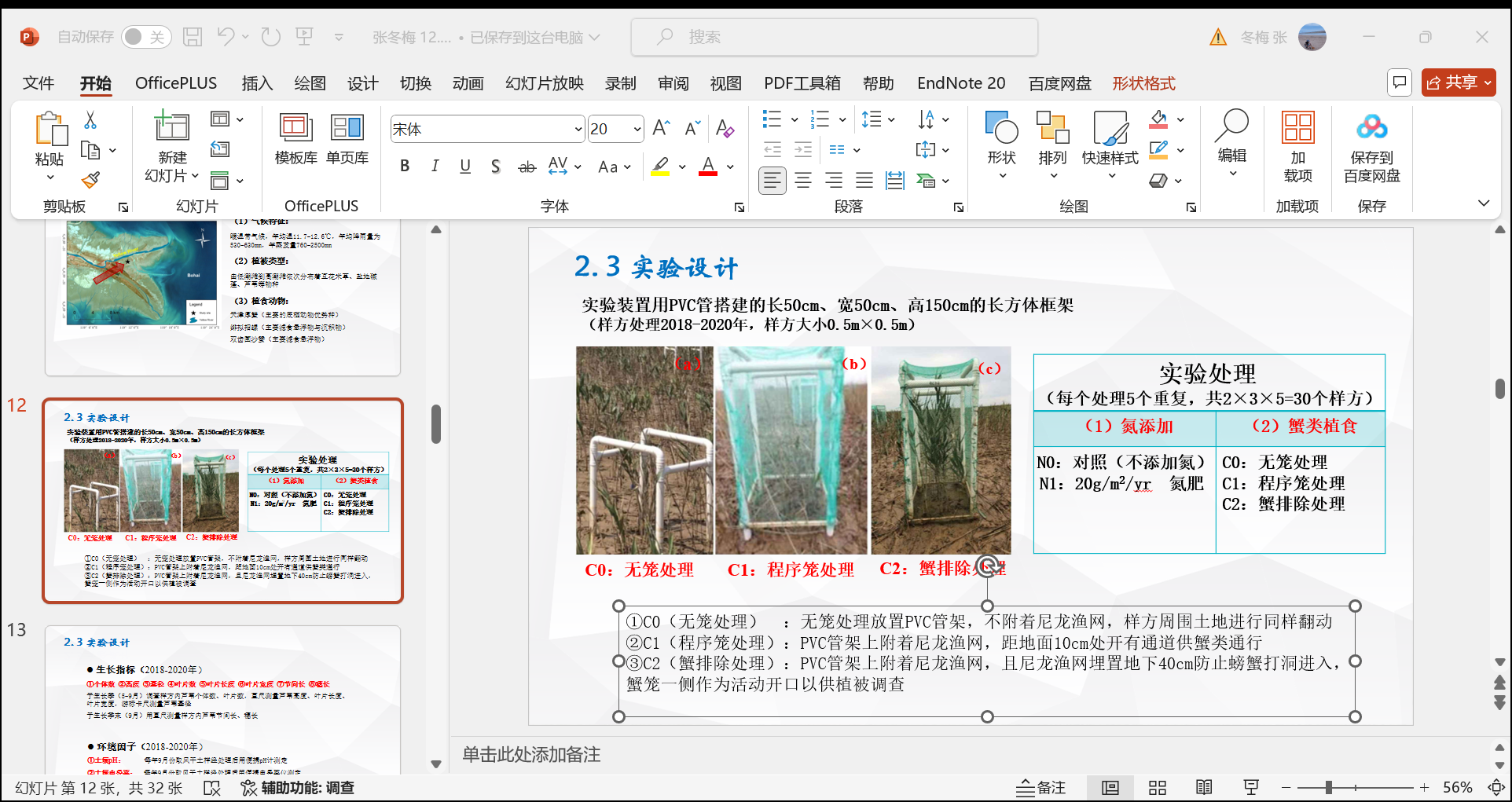
**

**Figure S2** Individuals of *P. australis* responding to Nitrogen Addition and Crab Treatment from2018 to 2020. (a) The effect of treatments on individuals in 2018; (b) The effect of treatments on individuals in 2019; (c) The effect of treatments on individuals in 2020.The red color box (N0C0) is No Nitrogen Addition and Ambient Crab treatment, the ginger box (N0C1) is No Nitrogen Addition and Procedural Crab Cage Treatment, the green box(N0C2) is No Nitrogen Addition and Crab Exclusion Cage Treatment, the lake blue box (N1C0) is Nitrogen Addition and Ambient Crab Treatment, the blue box (N1C1) is Nitrogen Addition and Procedural Crab Cage Treatment, and the pink one(N1C2) is Nitrogen addition and Crab Exclusion Cage Treatment.

**Figure S3** Height, leaf length and leaf breadth of *P. australis* responding to Nitrogen Addition and Crab Treatment in 2018 and 2019. (a) The effect of treatments on height in 2018; (b) The effect of treatments on height in 2019; (c) The effect of treatments on leaf length in 2018; (d) The effect of treatments on leaf length in 2019; (e) The effect of treatments on leaf breadth in 2018; (f) The effect of treatments on leaf breadth in 2019; The red color box (N0) is No Nitrogen Addition, the lake blue box(N1) is Nitrogen Addition. The letters in figure S3 indicate the multiple comparison results, the treatments with one same letter have no significant differences.

**Figure S4** The leaf number of *P. australis* responding to Nitrogen Addition and Crab Treatment from 2018 to 2020. (a) The effect of treatments on the leaf number of *P. australis* in 2018; (b) The effect of treatments on the leaf number of *P. australis* in 2019; (c) The effect of treatments on the leaf number of *P. australis* in 2020. Note: The red color box (N0C0) is No Nitrogen Addition and Ambient Crab treatment, the ginger box (N0C1) is No Nitrogen Addition and Procedural Crab Cage Treatment, the green box(N0C2) is No Nitrogen Addition and Crab Exclusion Cage Treatment, the lake blue box (N1C0) is Nitrogen Addition and Ambient Crab Treatment, the blue box (N1C1) is Nitrogen Addition and Procedural Crab Cage Treatment, and the pink one(N1C2) is Nitrogen addition and Crab Exclusion Cage Treatment.

**Figure S5** The length of *P. australis* internode responding to Nitrogen addition and Crab treatment from 2018 to 2020. (a) The effect of treatments on the *P. australis* internode in 2018; (b) The effect of treatments on the *P. australis* internode in 2019; (c) The effect of treatments on the *P. australis* internode in 2020. Note: The red color box (C0) is Ambient Crab Treatment, the green box (C1) is Procedural Cage Control Treatment, and the blue one(C2) is Crab Exclusion Treatment. The letters in figure S5 indicate the multiple comparison results, the treatments with one same letter have no significant differences.

**Figure S6** A linear correlation between biomass estimation and actual biomass measurement, with the abscissa being the estimated biomass and the ordinate being the measured biomass.

**Figure S7** The effects of Nitrogen Addition and Crab Treatment on the environmental factors. (a)The effects of treatments on the soil pH. The red color box (N0C0) is No Nitrogen Addition and Ambient Crab treatment, the ginger box (N0C1) is No Nitrogen Addition and Procedural Crab Cage Treatment, the green box(N0C2) is No Nitrogen Addition and Crab Exclusion Cage Treatment, the lake blue box (N1C0) is Nitrogen Addition and Ambient Crab Treatment, the blue box (N1C1) is Nitrogen Addition and Procedural Crab Cage Treatment, and the pink one(N1C2) is Nitrogen addition and Crab Exclusion Cage Treatment. (b)The effects of treatments on the soil electronic conductivity. The red color box (C0) is Ambient Crab Treatment, the green box (C1) is Procedural Cage Control Treatment, and the blue one(C2) is Crab Exclusion Treatment. (c)The effects of treatments on the total nitrogen content of soil. The red color box (N0C0) is No Nitrogen Addition and Ambient Crab treatment, the ginger box (N0C1) is No Nitrogen Addition and Procedural Crab Cage Treatment, the green box(N0C2) is No Nitrogen Addition and Crab Exclusion Cage Treatment, the lake blue box (N1C0) is Nitrogen Addition and Ambient Crab Treatment, the blue box (N1C1) is Nitrogen Addition and Procedural Crab Cage Treatment, and the pink one(N1C2) is Nitrogen addition and Crab Exclusion Cage Treatment. The letters in figure S7 indicate the multiple comparison results, the treatments with one same letter have no significant differences.

**Figure S8** Results of structural equation models on the consequences of Nitrogen Addition and Crab Treatment in 2019 (a) and 2020 (b). The green line indicates a positive correlation, the red line indicates a negative correlation, the solid line indicates direct influences, the width of the line and arrow indicates the strength of the relationship, and R^2^ indicates the proportion of the total variance explained by the model.
